# Intestinal epithelial barrier integrity investigated by label-free techniques in ulcerative colitis patients

**DOI:** 10.1038/s41598-023-29649-y

**Published:** 2023-02-15

**Authors:** Elsie Quansah, Elena Gardey, Anuradha Ramoji, Tobias Meyer-Zedler, Bianca Goehrig, Astrid Heutelbeck, Stephanie Hoeppener, Michael Schmitt, Maximillian Waldner, Andreas Stallmach, Jürgen Popp

**Affiliations:** 1 Institute of Physical Chemistry and Abbe Center of Photonics (IPC), Member of the Leibniz Centre for Photonics in Infection Research (LPI), Friedrich Schiller University Jena, Helmholtzweg 4, 07743 Jena, Germany; 2 Leibniz Institute of Photonic Technology (IPHT), Member of Leibniz Health Technologies, Member of the Leibniz Centre for Photonics in Infection Research (LPI), Albert-Einstein-Straße 9, 07745 Jena, Germany; 3 Department of Internal Medicine IV (Gastroenterology, Hepatology, Infectious Diseases and Interdisciplinary Endoscopy), Jena University Hospital, Friedrich Schiller University Jena, Am Klinikum 1, 07747 Jena, Germany; 4 Friedrich Schiller University Jena, Jena Center for Soft Matter (JCSM), Philosophenweg 7, 07743 Jena, Germany; 5 Jena University Hospital, Center for Sepsis Control and Care (CSCC), Friedrich Schiller University Jena, Erlanger Allee 101, 07747 Jena, Germany; 6 Institute for Occupational, Social, and Environmental Medicine, Jena University Hospital, Friedrich Schiller University Jena, Am Klinikum 1, 07747 Jena, Germany; 7 Laboratory of Organic and Macromolecular Chemistry (IOMC), Friedrich Schiller University Jena, Humboldtstraße 10, 07743 Jena, Germany; 8 Department of Medicine, University of Erlangen-Nuremberg, 91054 Erlangen, Germany

**Keywords:** Gastroenterology, Chemistry

## Abstract

The intestinal epithelial barrier, among other compartments such as the mucosal immune system, contributes to the maintenance of intestinal homeostasis. Therefore, any disturbance within the epithelial layer could lead to intestinal permeability and promote mucosal inflammation. Considering that disintegration of the intestinal epithelial barrier is a key element in the etiology of ulcerative colitis, further assessment of barrier integrity could contribute to a better understanding of the role of epithelial barrier defects in ulcerative colitis (UC), one major form of chronic inflammatory bowel disease. Herein, we employ fast, non-destructive, and label-free non-linear methods, namely coherent anti-Stokes Raman scattering (CARS), second harmonic generation (SHG), two-photon excited fluorescence (TPEF), and two-photon fluorescence lifetime imaging (2P-FLIM), to assess the morpho-chemical contributions leading to the dysfunction of the epithelial barrier. For the first time, the formation of epithelial barrier gaps was directly visualized, without sophisticated data analysis procedures, by the 3D analysis of the colonic mucosa from severely inflamed UC patients. The results were compared with histopathological and immunofluorescence images and validated using transmission electron microscopy (TEM) to indicate structural alterations of the apical junction complex as the underlying cause for the formation of the epithelial barrier gaps. Our findings suggest the potential advantage of non-linear multimodal imaging is to give precise, detailed, and direct visualization of the epithelial barrier in the gastrointestinal tract, which can be combined with a fiber probe for future endomicroscopy measurements during real-time in vivo imaging.

## Introduction

Inflammatory bowel diseases (IBD) are complex chronic diseases of the gastrointestinal tract with an unclear etiology. The number of patients with IBD, including Crohn’s disease and ulcerative colitis (UC), is dramatically increasing worldwide, posing a global public health challenge. Genetic predispositions, the environment, and microbiome-host interactions play a crucial role in the pathogenesis of IBD^1–3^. Moreover, intestinal barrier dysfunction significantly contributes to the pathophysiology of IBD^4–7^.

While the healthy epithelium is composed of a monolayer of intestinal epithelial cells connected by an apical junction complex (AJC)^8^, structural alteration in the AJC causes inappropriate permeability during inflammation. Additionally, apoptosis and lesioning of epithelial cells are implicated in intestinal barrier dysfunction. Subsequent processes, such as the penetration of luminal antigens and bacteria, induce a cascade of immune reactions in the lamina propria that further increase the damage to the intestinal barrier^9,10^. The disruption of the mucus layer can be a trigger for the penetration of luminal antigens and favor the development of UC^11^. In general, a damaged intestinal epithelial barrier, depletion of the mucus layer, altered tight junctions and adherens junctions, increased paracellular permeability, and the creation of epithelial gaps have all been identified in IBD^10,12^.

Consequently, various methods have emerged for the assessment of mucosal inflammation. To date, ileocolonoscopy with endoscopic biopsies and pathological examination remain the gold standard. Both methods have proved useful in detecting dysplasia and the severity of diseases, but the procedure is not label-free, is time-consuming, and lacks sufficient spatial resolution for detecting minute tissue alterations. In addition, methods such as computed tomography, ultrasound, and magnetic resonance imaging have been established as non-invasive imaging techniques for pathological investigations^13–15^. Although these techniques have succeeded in the detection of mucosal inflammation, limited spatial resolution restricts visualization of the epithelial barrier defects and requires further research to improve their diagnostic potential^16^.

To integrate endoscopy with microscopy, an endomicroscopic technique such as confocal laser endomicroscopy (CLE) has been utilized in molecular imaging of the gastrointestinal tract. This technique is currently being used in clinical studies because it provides real-time detection up to sub-cellular resolution^17^. Kiesslich et al.^5^ showed local barrier dysfunction and permeability defects using confocal endomicroscopy with intravenous fluorescein. The drawback of this endomicroscopy method is the intravenous application of contrast agents immediately before imaging. Concerns have been raised about the ability of contrast agents to induce genetic mutations in DNA^18^. Moreover, Yan et al.^19^ compared multi-photon and CLE imaging and reported that the intravenous administration of fluorescent agents resulted in blurred images, which eventually affected the accuracy of the diagnosis. Other techniques, such as light-sheet microscopy, fluorescence microscopy, electron microscopy, etc., have high resolution but are limited to ex vivo examinations^20–22^.

Emerging biophotonic methods that address these limitations of CLE are non-linear multimodal techniques, such as coherent anti-Stokes Raman scattering (CARS), second harmonic generation (SHG), two-photon excited fluorescence (TPEF), and two-photon fluorescence lifetime imaging (2P-FLIM). These methods offer increased tissue penetration depth due to the use of a near-infrared laser, provide subcellular resolution, and help in analyzing the cell’s metabolic states without the use of external labels^23–26^. The additional benefit of label-free non-linear multimodal imaging over conventional histopathology is improved morpho-chemical contrast in non-linear images. CARS visualizes the lipid distribution, TPEF images the intrinsic autofluorophores, SHG specifically highlights the distribution of collagen within the tissue, and 2P-FLIM provides chemical contrast based on the lifetime information of fluorophores. Moreover, the potential of multimodal imaging to be translated to fiber-based in vivo imaging while preserving the tissue’s integrity makes it an invaluable approach for biomedical diagnosis.

To the best of our knowledge, this study presents, for the first time, direct visualization of intestinal epithelial barrier gaps in the inflamed colonic mucosa biopsied from UC patients using label-free non-linear multimodal imaging methods. The results presented focused on the identification of barrier defects and the characterization of tissue alterations between the control and inflamed colonic mucosa, collected from healthy donors and UC patients, respectively. The findings of the imaging methods are supported by transmission electron microscopy (TEM) performed to assess the apical junction complex. The direct detection of epithelial defects based on visual inspection without the need for staining agents and rigorous image analysis algorithms is an added advantage, and when combined with endomicroscopy^27^, it offers high diagnostic potential for a better understanding of pathophysiology in inflammatory diseases.

## Results

To demonstrate the potential of non-linear multimodal imaging for detailed visualization of epithelial barrier destruction, the colonic mucosal biopsies from patients with mild-moderate inflammation (histological index of Mayo subscore I–II) and moderate-severe inflammation (Mayo subscore II-III) were compared with the colonic mucosal biopsies from healthy individuals.

**Figure 1:**
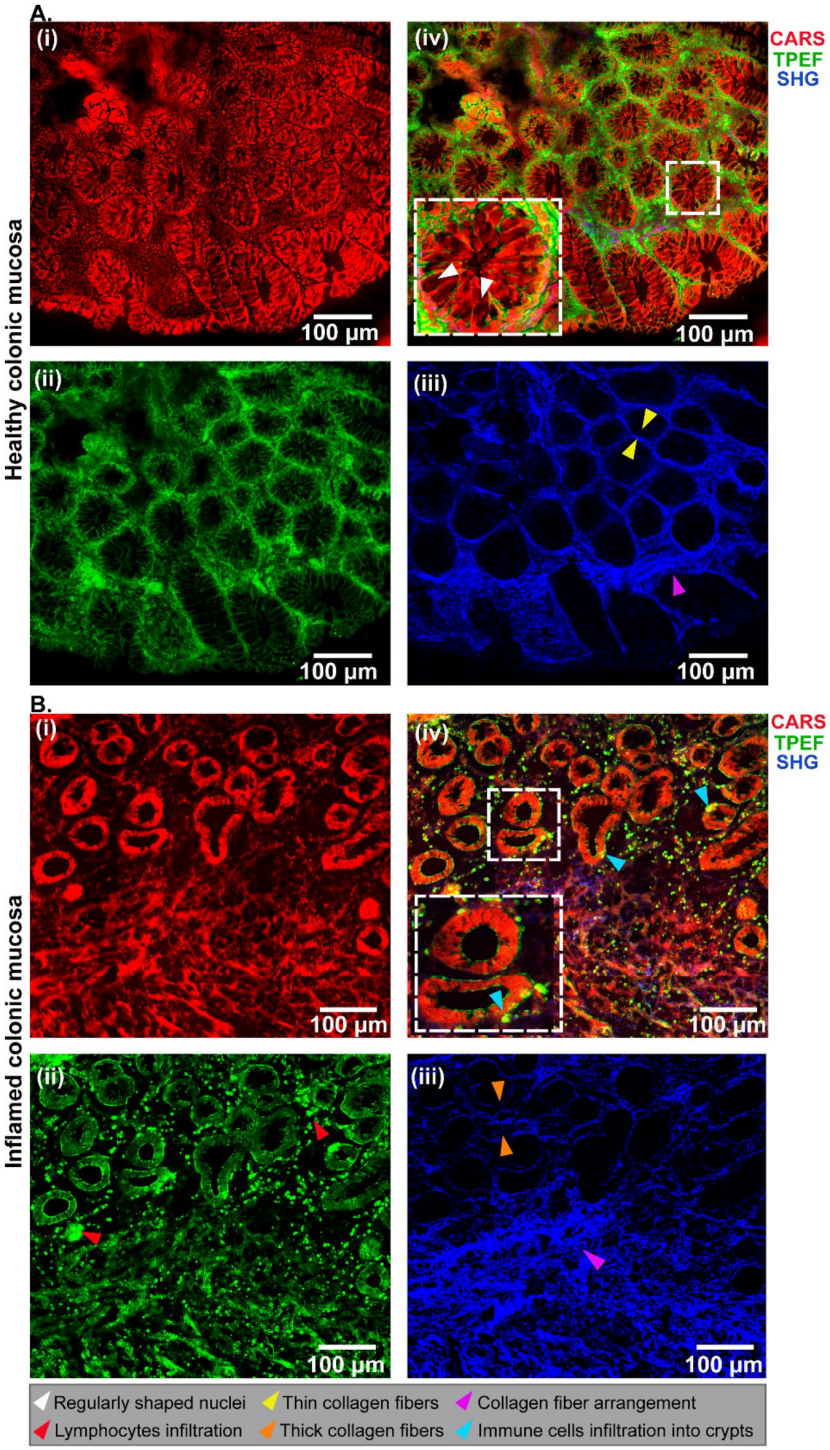
Non-linear multimodal images of healthy (**A**) and inflamed (**B**) colonic mucosa with a combination of CARS @ 2850 ${\mathrm{cm}}^{-1}$ (**Ai and Bi**), TPEF @ 458bp64 (**Aii** and **Bii**), and SHG @ 415bp3 (**Aiii** and **Biii**). The composite images contain all three channels (**Aiv** and **Biv**), and the inset shows zoomed-in regions of interest, which are indicated by white dashed squares. The arrowhead labels are indicated below the images.

The multimodal images (Fig. 1) provide a detailed outline of the colonic mucosa, with intestinal epithelium that forms crypts and the lamina propria. The intestinal epithelium forms a cell layer on the luminal surface with homogeneously distributed crypts (Fig. 1Ai–Aiv), in contrast to the inflamed tissue (Fig. 1Bi–Biv). In the inflamed tissue, a distortion in the crypt structure, crypt branching resulting in some merged crypts, and increased variability in the inter-cryptal distance (Fig. 1B) were apparent defects visible in the architecture of the biopsies from patients with mild-moderate inflammation^28^.

The morpho-chemical information derived from the multimodal images allows the assessment of intrinsic endogenous markers in the tissue section. For instance, the red pseudo-color highlights the distribution of the lipids captured by the CARS signal of methylene groups at 2850 cm^−1^. The lipid signal is prominent in the healthy colonic mucosa (Fig. 1Ai) compared to the severely inflamed tissue (Fig. 1Bi), indicating the role of lipid metabolism in the pathogenesis of intestinal inflammation^29^. The TPEF channel (in green, Fig. 1Aii) primarily exhibits NAD(P)H autofluorescence, which is one of the most abundant autofluorescent molecules found in cells and tissues, making it an excellent indicator of a cell's metabolic state^30^. According to Ghukasyan et al.^31^, the fluorescent emission of NAD(P)H ranges between 440 and 470 nm when excited at a wavelength between 330 and 360 nm. These excitation and emission wavelengths are comparable to those used in our study (i.e., 336 nm and 465 nm).

Unlike in the healthy colonic mucosa, an increase in the TPEF signal was observed as strong autofluorescence indicated by bright green cells (red arrows in Fig. 1Bii). Furthermore, the production of reactive oxygen species by macrophages during inflammation promotes the synthesis of NAD(P)H^32,33^, which explains the strong green signal of NAD(P)H observed in IBD patient samples. In the case of SHG imaging (Fig. 1Biii), the lamina propria reveals collagen fibers (displayed in blue) surrounding the crypts to give structure to the cellular arrangement.

Orderly organized and thin collagen fibers were observed in the normal colon samples (Fig. 1Aiii), while the inflamed tissues had a thicker network of collagen fibers. In contrast to this, the proper arrangement of the collagen fibers was lost within the muscularis mucosa region in Fig. 1Biii.

**Figure 2:**
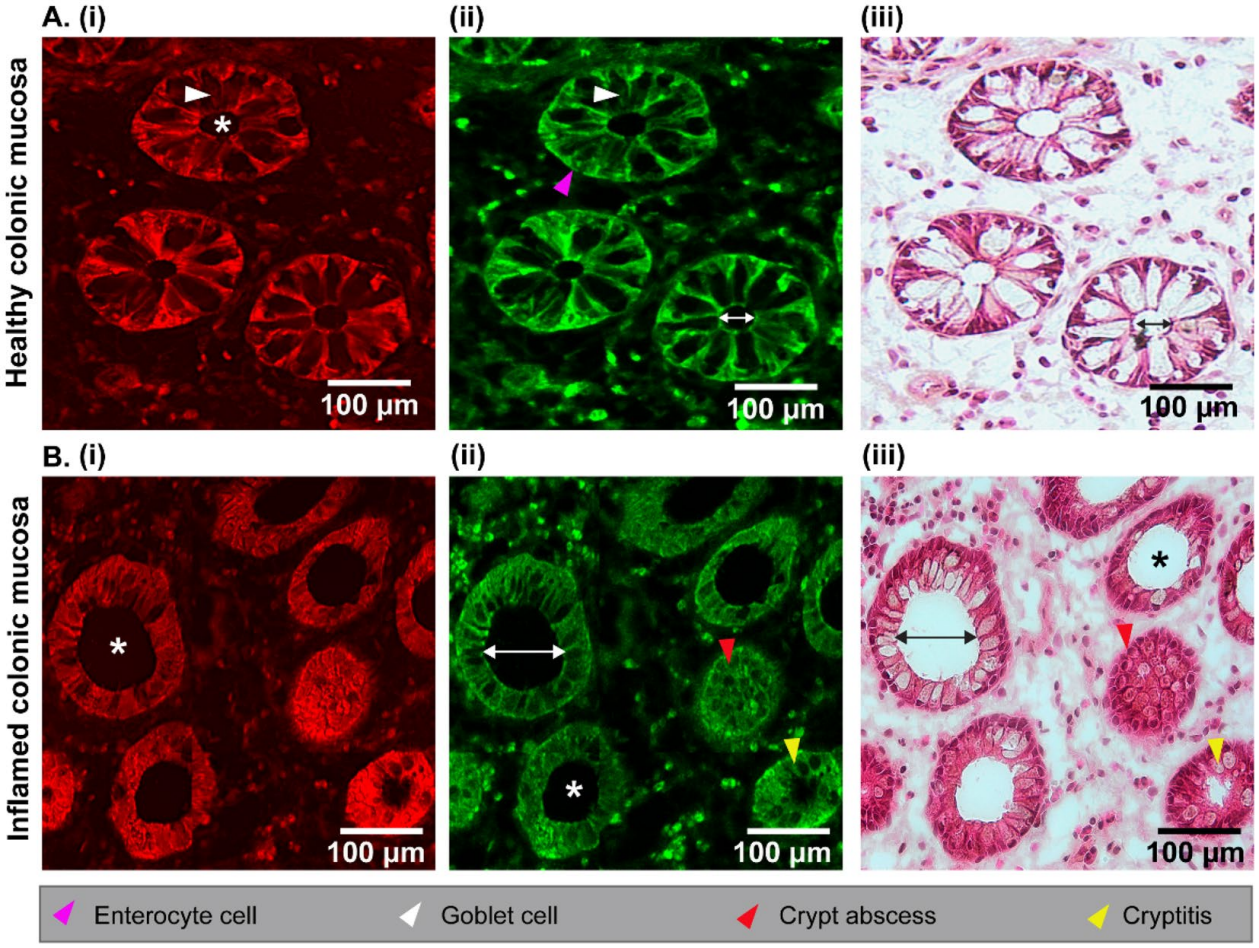
Comparison of multimodal and H&E images of healthy (**A**) and inflamed (**B**) colonic mucosa. The CARS (**Ai, Bi**) and TPEF (A**ii** and **Bii**) images in comparison with H&E stained (**Aiii** and **Biii**) images. The asterisks (*) indicate intact and enlarged cryptal lumen regions for healthy and inflamed colonic mucosa, respectively. Arrowhead labels are indicated below the images, and the double-sided arrows measure the lumen diameter for healthy (~ 22 μm) and inflamed colonic mucosa (~ 103 μm).

To prove that multimodal imaging is useful in biomedical applications, it must be compared with the gold standard for medical diagnosis. Here, the multimodal images were correlated with hematoxylin and eosin (H&E) stained images of the same tissue sections. In agreement with the histopathological analysis (Fig. 2Aiii and Biii), both the CARS (Fig. 2Ai and Bi) and TPEF (Fig. 2Aii and Bii) images showed dark structures of the goblet cells due to mucin in secretory vesicles. This enables a clear distinction between enterocyte cells in the TPEF channel (pink arrow) and goblet cells (white arrows in Fig. 2A) in a label-free manner, as reported in the study by Chernavskaia et al.^34^. The intact intestinal epithelium was observed in healthy colon tissue sections as well as intact crypt lumen diameters of approximately 22 μm. In contrast to the healthy colon, major indicators of inflammation from Fig. 2B, including an increase in the crypt lumen diameters (~ 103 μm) and a slightly changing crypt axis with a change in goblet cell density, were recognized in inflamed mucosa (Fig. 2Bi–Biii). In addition, cryptitis and crypt abscess were noticeable in TPEF (Fig. 2Bii) and the corresponding histological images (Fig. 2Biii). A crypt abscess is a typical occurrence in UC patients where the crypt lumen is infiltrated by neutrophils, which tend to migrate into the crypt epithelium. This can also result in disintegration and rupture of the epithelial cells^35–37^.

**Figure 3:**
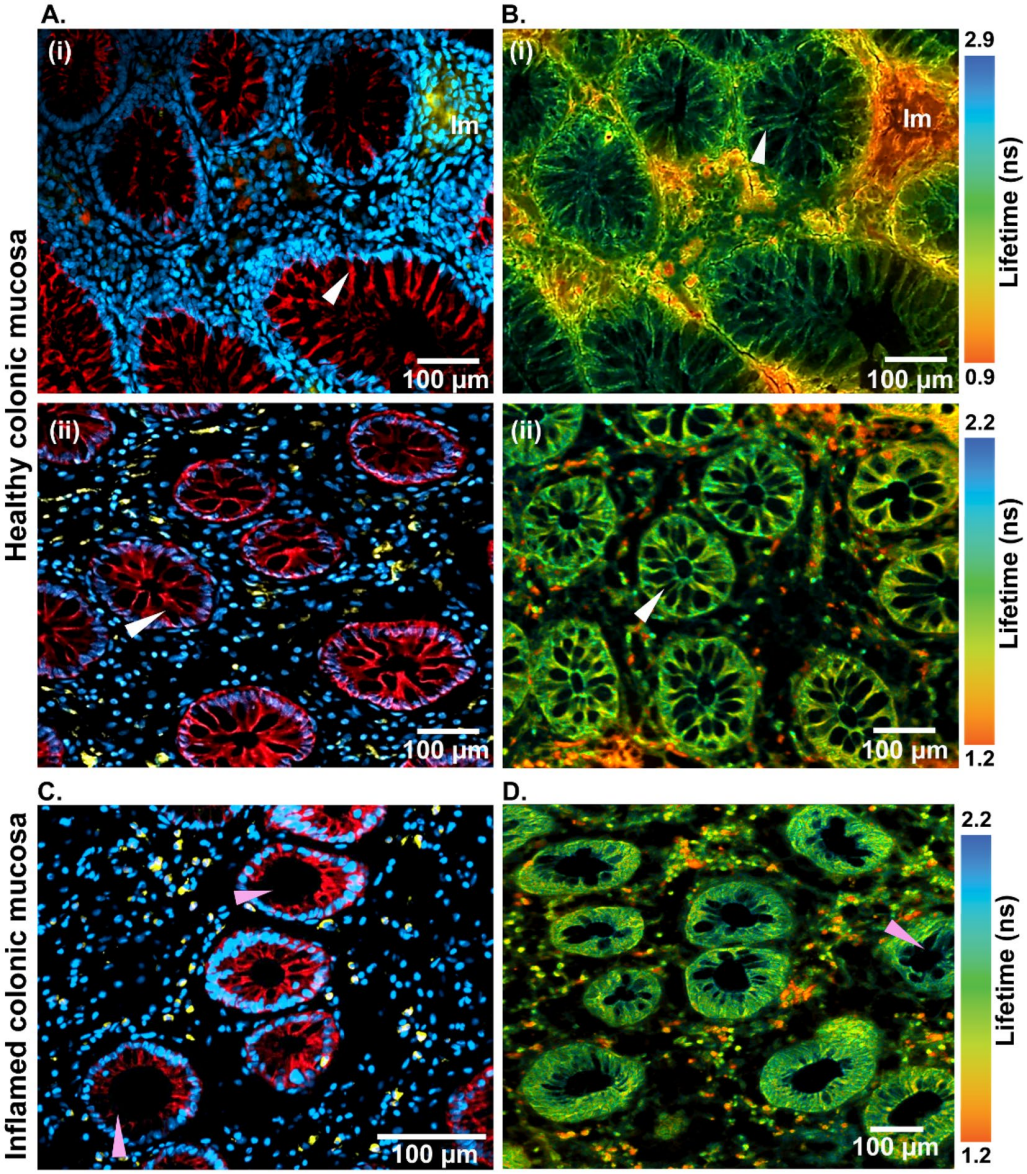
Immunofluorescence (**A**, **C**) and FLIM (**B**, **D**) images of healthy (**A**, **B**) and inflamed (**C**, **D**) colonic mucosa. The immune cells (Im) from the immunofluorescence images (**Ai**) were correlated with the FLIM images (**Bi**) through their autofluorescence lifetime. Intact epithelial barriers were identified in the healthy colonic mucosa as shown by white arrows (**Aii**, **Bii**), while the loss of barrier integrity was indicated by pink arrows. A false color-coded representation of mean fluorescence lifetime in the range of 0.9–2.9 ns (**Bi**) and 1.2–2.2 ns (**Bii**, **D**) has been shown in FLIM images. The immunofluorescence staining is shown in blue (DAPI): nuclei, red (E-cadherin): epithelium, and yellow (CD11b): immune cells.

Additionally, damage to the epithelial barrier and infiltration of immune cells into the crypts were identified in the inflamed colonic mucosa, along with compromised E-cadherin junctions. The neutrophil infiltration into the epithelium was clearly visible (Fig. 3C), highlighted by the CD11b stain and depicted in pseudo-yellow color. These changes were further investigated via immunohistochemistry (IHC). The high-resolution information revealed by the IHC (Fig. 3A and C), such as the apical junction complex (connecting the epithelial cells) and immune cell infiltration, was retrieved by 2P-FLIM (Fig. 3B and D), by investigating the colonic mucosa in its native state without using any external labels or sophisticated analysis methods. To exploit the advantage of FLIM over stained images, optical fingerprinting could be performed in future research to selectively identify and extract individual immune cells based on their lifetime information.

Overall, the structural defects of the inflamed tissue observed by the inexpensive, non-laborious, and fast multimodal imaging techniques are in good agreement with the histopathological analysis (Fig. 2) and the classical immunostaining method (Fig. 3).

In contrast to the IHC, 2P-FLIM provides morpho-chemical contrast based on intrinsic endogenous markers and the changes due to metabolic activities. It has proved useful in several biomedical applications as a gastrointestinal diagnostic tool for analyzing mucosal lifetime changes and monitoring the treatment of inflammatory diseases^38–40^.

Here, image contrast was obtained from the NAD(P)H autofluorescence using lifetime information to differentiate epithelial cells from immune cells (Fig. 3C and D). Both imaging techniques could identify intact epithelial borders in the healthy colonic mucosa, as indicated by white arrows. In the IHC images, E-cadherin stain specifically highlighted the architecture of the epithelial barrier (Fig. 3Ai and Aii), and 2P-FLIM detected the preserved epithelial structure with high chemical contrast in a label-free manner (Fig. 3Bi and Bii).

**Figure 4:**
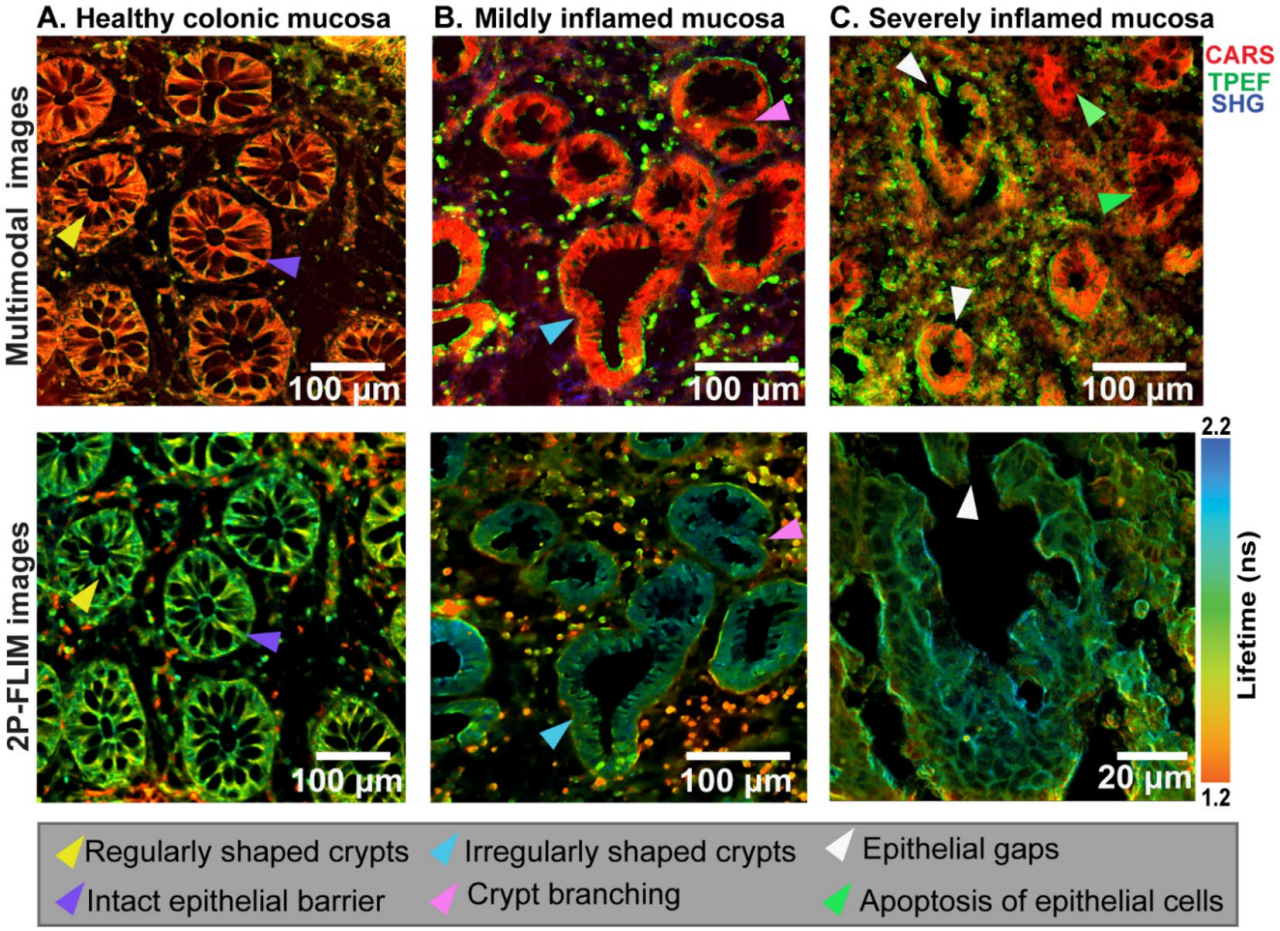
Non-linear multimodal imaging of healthy colonic mucosa (**A**), mildly inflamed mucosa (Mayo subscore I–II) (**B**), and severely inflamed mucosa (Mayo subscore II–III) (**C**). The CARS/TPEF/SHG composite images containing all three channels are displayed in the upper row, and the 2P-FLIM images are shown in the lower row. The arrowhead labels describing the epithelium barrier integrity are indicated below the images.

In Fig. 4, the multimodal images of colon tissue from patients with different stages of mucosal inflammation severity have been compared with healthy colon tissue. The CARS/TPEF/SHG and 2P-FLIM images collected in a co-registered manner show the transition from an intact epithelial border in the healthy tissue (Fig. 4A) to a compromised epithelial border in the mildly inflamed colonic mucosa (Fig. 4B), and evidence of an epithelial barrier defect via epithelial gap formation in the severely inflamed mucosa (Fig. 4C). Moreover, 2P-FLIM was measured because, unlike intensity-based TPEF measurements, it can distinguish fluorophores with overlapping spectral characteristics at high chemical contrast. Specifically, the immune cells in the 2P-FLIM images showed differing lifetime information (pseudo-colored in red-to-green) compared to the immune cells in the TPEF images, depicted only in green color in the upper row of the multimodal images (Fig. 4B).

The epithelial barrier defects and epithelial discontinuities were directly visualized without the need for machine learning algorithms. The extent of barrier defects increases with an increase in the disease severity, i.e., from moderate to severe inflammation. In the colon tissue of severely inflamed mucosa, “epithelial gaps” formation is distinctly visible (Fig. 4C), which could probably be due to the apoptosis of enterocytes^41,42^. These epithelial gaps lead to compromised intestinal permeability, a hallmark of inflammatory bowel diseases^6^.

### Epithelial gap formation in severely inflamed colonic mucosa

In a healthy colonic mucosa, cell shedding is considered a normal phenomenon that causes the formation of epithelial gaps. However, epithelial cells extend protrusions beneath shedding enterocytes, and the junction proteins that are formed by neighboring cells seal the gap^43^. During inflammation, several cells are shed at the same time, resulting in epithelial discontinuities. As a result, barrier integrity is compromised because proper intercellular adhesion does not occur^44–46^. Other studies have also hypothesized that gap formation is caused by the detachment of the junctions^47^.

According to previous research, confocal endomicroscopy (CLE) provides a significant characterization of epithelial gaps and local barrier dysfunction in UC patients by administering fluorescein^45^. Although CLE has played a crucial role in intravital gastrointestinal diagnosis, it still relies on the use of fluorescent contrast agents and offers limited tissue penetration depth. In contrast, 3-dimensional (3D) multimodal imaging can give an enhanced visualization of the entire volume of the mucosal crypt in a label-free manner. Therefore, for detailed analysis and evaluation of the epithelial gaps in the crypts, multimodal label-free imaging was performed on 20 μm tissue sections of the human mucosal biopsies from three severely inflamed UC patients.

**Figure 5:**
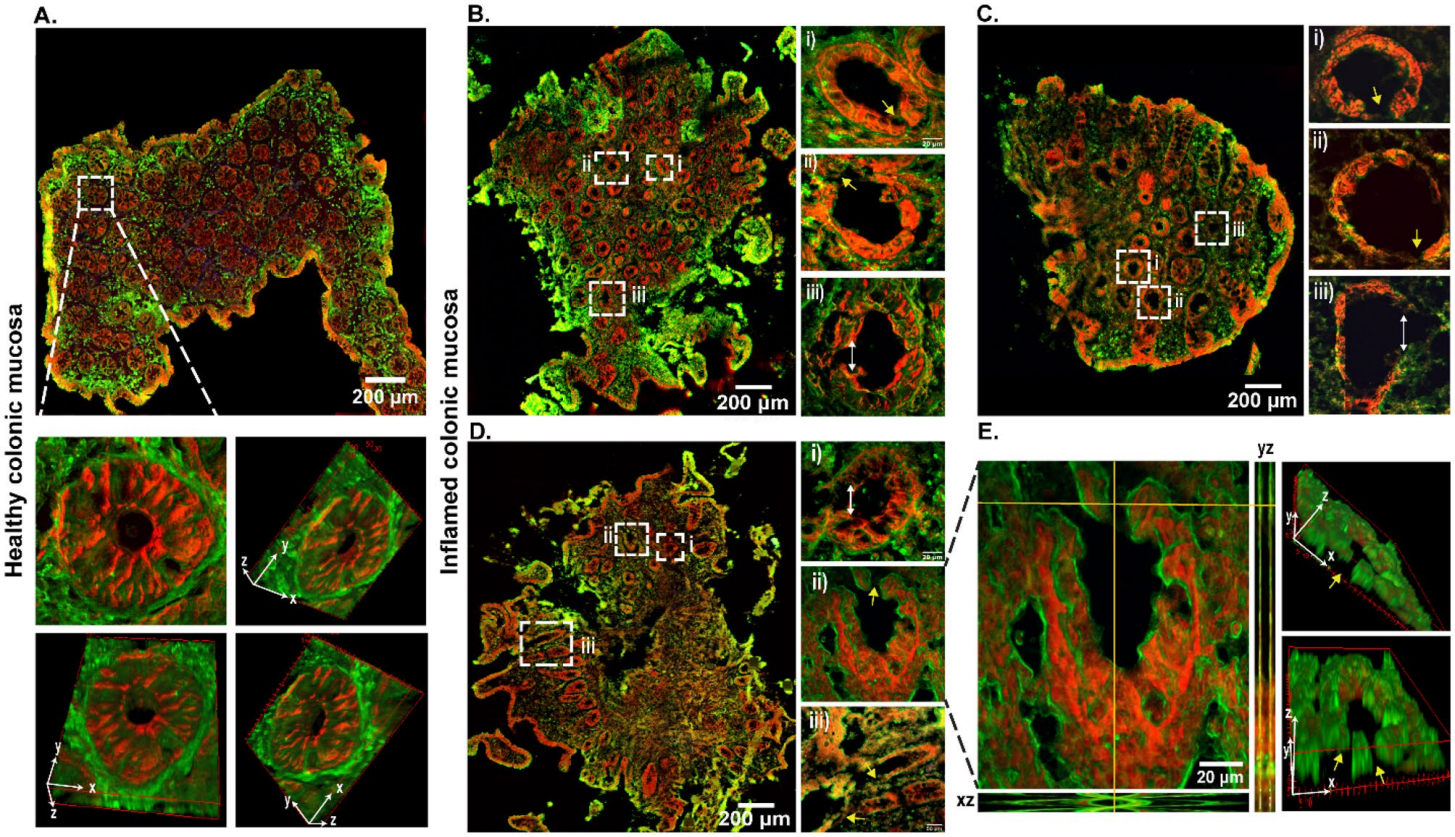
Epithelial gap detection of healthy mucosal tissues (**A**) and severely inflamed tissues by non-linear multimodal imaging of CARS (red channel) and TPEF (green channel). Overview images of the whole inflamed tissue cryosection from patient 1 (**B**), patient 2 (**C**), and patient 3 (**D**), along with the zoomed regions of interest in the respective insets (**i, ii, iii**). Orthogonal views are also displayed in the xz and yz directions to reveal gap formation (**E**), as well as volume measurements of the 3D view to show gaps at different orientations. Yellow arrows indicate the gaps as breaks in the epithelial barrier, and the white arrow depicts the length of the gap. For clarity, the respective insets marked in the tissue from each patient have been zoomed in (patient 1: Fig. 5Bi–Biii; patient 2: Fig. 5Ci–Ciii; patient 3: Fig. 5Di–Diii). The orthogonal views in the xz and yz-axis further provide evidence of barrier loss, demonstrating the presence of gaps within all focal planes as well as 3D views at different orientations (Fig. 5E).

Figure 5 represents the tissue section from each patient sample compared to a healthy control sample. From Fig. 5A, the healthy tissue showed no indication of gaps as observed in various 3D orientations. Analogous to this, in inflamed tissue different regions were observed as discontinuities in the epithelial lining opening into the lumen, as indicated by the yellow arrows in Fig. 5B–E. The findings from the comparison of healthy and inflamed tissues confirm that epithelial gap formation is not a result of artifacts from sample preparation or the imaging process.

An attempt was made to qualitatively assess the epithelial gaps observed in the inflamed tissue. The epithelial gaps were classified by measuring the length of the gap from one end of the epithelial break to the other, as indicated by white arrows (see zoomed images in Fig. 5Biii, Ciii and Di). The total epithelial gap was calculated by measuring the length of all the observed gaps and summing them up to give the total gap length. For instance, in Fig. 5B, the total length of the epithelial gaps in the entire tissue cryosection was 130 μm. This, however, gave the lowest mean gap of 16 μm, since smaller gaps were observed. In the case of the cryosection in Fig. 5C, the total gap length was calculated to be 110 μm. Despite being the shortest recorded gap length, it produced the highest mean gap of 22 μm. This is because the tissue had fewer crypts but much wider gaps. The total length of the tissue section in Fig. 5D gave the highest gap length of 193 μm and a mean gap of 19 μm. Since the size of an epithelial cell ranges from 8 to 21 μm^48^, the gap could be formed by the loss of epithelial cells, as already discussed above. Therefore, quantification of the epithelial gap length indicates the degree of barrier defect per tissue section and can be further analyzed to indicate the severity of the epithelial damage.

### Analysis of intestinal epithelial barrier integrity by TEM

To have a closer look at the disruption of intestinal epithelial barrier integrity and support our results, transmission electron microscopy (TEM) was performed. The TEM images were stitched together to demonstrate a larger area of tissue samples, as seen in Fig. S1A for healthy colonic mucosa and Fig. S1B for inflamed colonic mucosa. The normal and well-preserved structure of the healthy colonic mucosa can be observed in Fig. S1A. Moreover, a high number of goblet cells (GC) are present in healthy human mucosa, which is in agreement with the previously shown CARS and TPEF images in Fig. 2, where mucin in secretory vesicles of goblet cells was recognized as dark structures. In contrast, the colonic mucosa from a patient with UC showed a reduced number of goblet cells (Fig. S1B), which ultimately leads to a limited mucus layer, compromising the barrier functions as well.

**Figure 6:**
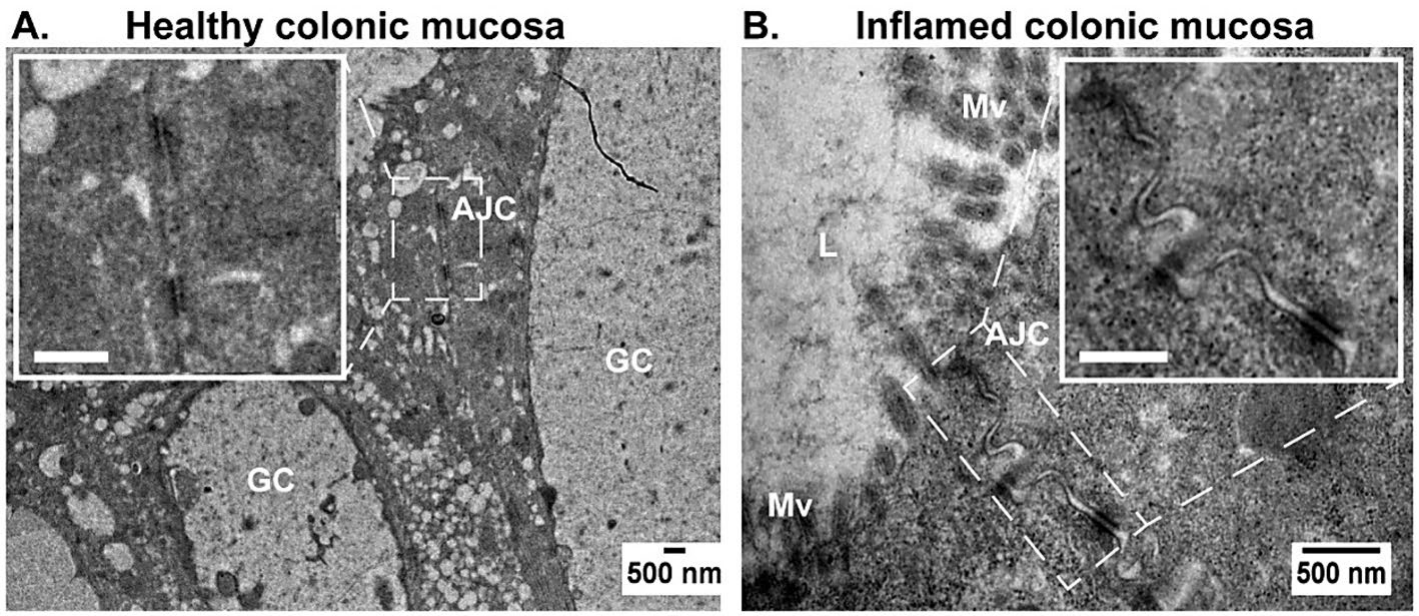
Transmission electron microscopy (TEM) images of healthy (**A**) and inflamed (**B**) colonic mucosa. The apical junction complex (AJC) is visible between two epithelial cells and is represented by dashed lines. The inset shows a zoomed-in region of interest, which is indicated by a white square with a scale bar corresponding to 500 nm. Mv, microvilli; GC, goblet cells; L, lumen.

Considering that the apical junction complex (AJC) regulates intestinal epithelial barrier integrity^9,49^, the alteration of the AJC in inflamed colonic mucosa is key to determining barrier defects. Here, the TEM images could visualize the AJC in both healthy and inflamed mucosa. In Fig. 6A, a structured AJC can be recognized between epithelial cells of the healthy colonic mucosa and can be seen more clearly in the zoomed-in inset. In contrast, the AJC between the epithelial cells of inflamed mucosa is disturbed and characterized by increased intercellular spaces (Fig. 6B). The disruption of intestinal epithelial barrier integrity can be recognized as well via the noticeable changes in microvilli (Mv) (Figs. 6B, S2C, and S2D).

The microvilli of epithelial cells in healthy colonic mucosa are intact (Fig. S2A and S2B). However, there is a clear difference in the microvilli of inflamed mucosa compared with normal tissue. In Fig. S2C and S2D, the microvilli are disturbed, shorter, and thicker than in the healthy sample.

It was crucial to perform TEM to investigate epithelial barrier damage to support our 3-dimensional (3D) data. Using electron microscopy, the cell structures are usually visible in the tissue. However, given the high resolution of TEM, it was challenging to capture the image of crypts with epithelial gaps. Nevertheless, there were noticeable changes in the epithelial barrier of the colonic mucosa of the UC patient with the destruction of the AJC, disturbance of microvilli, and the lack of goblet cells (Fig. S2C and S2D). It is important to mention that the majority of the inflamed tissue was destroyed, and the clear structure of the mucosa was rarely found in our samples. The detailed analysis of TEM images supports our non-linear multimodal data on intestinal epithelial barrier integrity disruption in patients with UC. We demonstrated the clear and well-preserved structure of the healthy colonic mucosa and noticeable changes in the epithelial barrier in inflamed colonic mucosa.

## Discussion

The limitations of currently used confocal laser endomicroscopy (CLE) desires for new alternatives that would help to diagnose the alterations of intestinal epithelial barrier integrity.

In this contribution, the capability of non-linear multimodal imaging to evaluate the epithelial barrier defects in ulcerative colitis patients was addressed. The combination of CARS, TPEF, SHG, and 2P-FLIM provided distinct morpho-chemical information about the colonic mucosa and presented a direct visualization of tissue alterations without the need for any external stains or sophisticated image analysis algorithms. Endogenous markers such as lipids, NAD(P)H, and collagen were used to highlight the tissue alterations and distinguish between the intact epithelial barrier in the healthy colonic mucosa and a compromised barrier in the inflamed colonic mucosa.

Subsequent investigations on co-registered images were performed based on histological and immunofluorescence imaging. A good correlation was found between multimodal imaging and histological imaging, validating the potential of non-linear imaging for clinical applications. Though similar morphological information was extracted from both imaging methods, specific chemical information such as the overall lipid intensity or the fluorophore and immune cell characterization could not be retrieved by histological imaging. This meaningful image contrast is backed by a fast and accurate diagnosis for more detailed information on ulcerative colitis tissue alterations.

Additional information was provided by the immunofluorescence imaging, as the inflamed colonic mucosa demonstrated distorted E-cadherin junctions. This is a necessary part of the research since E-cadherin plays an essential role in the maintenance of the intestinal barrier function^50^ for intercellular adhesion^51^, and usually, because transmembrane proteins are useful for visualization of tissue architecture.

We proved in this context the transition of intestinal epithelial integrity in a healthy colonic mucosa, a mildly inflamed mucosa (Mayo-score I-II), and finally, a severely inflamed mucosa (Mayo-score II–III). The mildly inflamed mucosa highlighted various occurrences that could contribute to epithelial barrier defects. Amongst these were cryptitis, crypt abscess, immune cell infiltration into crypts, increased crypt luminal orifice, and altered E-cadherin junctions. However, a clear indication of epithelial barrier damage was identified in the severely inflamed colonic mucosa, as the formation of epithelial gaps in the lining of the epithelium was observed. Further 3D analysis was performed to give a detailed overview of the entire epithelial volume. From this finding, the gap formation was indicative of the fact that apoptosis of enterocytes could have occurred, as the average size of the gaps was found to be in the range of epithelial cells. Another possibility for the break in the epithelium could be a damaged tight junction or an uncontrolled rate of cell shedding without replenishment^52^.

Complementary studies of TEM revealed the disturbance of the apical junction complex, characterized by an increased intercellular space in the inflamed tissue. Following this observation were other structural modifications, such as damaged microvilli and reduced goblet cells. The latter indicated the vulnerable state of the protective mucosal barrier in the mucus layer^10^.

In a nutshell, we have demonstrated the possibility to screen for the formation of epithelial gaps, disturbed AJC, and disruption of the epithelial barrier in ulcerative colitis patients using label-free non-linear imaging, supported by TEM.

## Conclusion

The presented work is, to our knowledge, the first to provide an ex vivo study that thoroughly investigates the intestinal epithelial barrier gaps and tissue damage in ulcerative colitis patients using label-free non-linear multimodal imaging techniques. Multimodal images enabled direct visualization and, without the need for any sophisticated data analysis procedures, the comparison of microscopic tissue alterations in inflamed colonic mucosa to normal mucosa from healthy donors and successful characterization of epithelial gaps found in crypts. Since the discontinuities in the intestinal mucosal barrier could be an indication of barrier disruption, the non-linear 3D analysis was performed, and the results were supported by TEM imaging to confirm the compromise of the apical junction complex and structural changes in the tissue.

Overall, the results presented here emphasize the novelty of non-linear techniques for fast diagnosis and evaluation of epithelial disruption in patients with UC, especially when integrated into colonoscopic investigations. This study opens a potential doorway for in vivo applications in combination with the previously shown fiber-based CARS/TPEF/SHG imaging setup for an endomicroscopic imaging probe. Hence, it offers the possibility of assessing label-free, high-resolution morpho-chemical information in vivo, in situ, without taking any biopsies.

## Methods

### Human colon biopsies

In this work, mucosal biopsies were collected from the sigmoid colon or the rectum during the routine colonoscopy of patients with UC (n = 8) at Jena University Hospital. Healthy individuals (n = 6) undergoing screening colonoscopy represented the control group. The study was approved by the committee of human ethics (3285-10/11) Jena University Hospital, Germany and the research performed are in accordance with relevant guidelines/regulations and as per the Declaration of Helsinki. All donors gave informed consent to participate in this study. The tissue sections from healthy individuals without any inflammation in the gastrointestinal tract served as controls. The state of inflammation in patients with UC was determined by endoscopic and histopathological features, using the histological index endoscopic Mayo subscore (0–III)^53,54^. The Mayo subscore I–II, i.e., mild-moderate inflammation, and Mayo subscore II–III, i.e., moderate-severe inflammation, were determined for the UC patients that were used for our study.

### Tissue sampling

The sample preparation was the same as mentioned previously^55^. Briefly, biopsies were immediately transferred from the endoscopy department to the laboratory within 5–7 min in previously oxygenated ice-cold modified Krebs–Ringer bicarbonate buffer. The biopsies were unfolded under a stereomicroscope and the tissues were fixed with Histoacryl Tissue Glue (BBraun, Spain) on a plastic disk (220 µm). The tissue samples were transferred to 4% paraformaldehyde with 25 mM glycine and replaced with a sucrose solution overnight. After fixation, biopsies were frozen in Tissue-Tek OCT Compound (Sakura, Japan) and sectioned by a cryotome (Leica CM 1050, Ireland) into 6 µm slices for staining and 20 µm slices for 3D analysis. 4% glutaraldehyde was used to fix the tissue for transmission electron microscopy (TEM) imaging.

### Non-linear multimodal imaging

The colon tissue samples were measured using a home-built non-linear multimodal setup. The instrumental details have been described in a previous publication^56^. Briefly, the laser source consists of a picosecond Ti: sapphire laser (Mira HP, Coherent, Santa Clara, CA, USA), which is split into two fractions with a beam splitter. One fraction is used with no frequency conversion, at a wavelength of 832 nm, as it serves as the CARS Stokes beam. The second fraction is coupled into an optical parametric oscillator (OPO, APE, Berlin, Germany) and converted into a wavelength of 672 nm, which is used as the CARS pump beam. The chosen wavelength difference of the pump and Stokes beam matches the wavenumber position of the CH_2_ stretching vibration at 2850 cm^−1^ displayed in the CARS images. An overlap of both pump and Stokes beams in space and time is required for CARS imaging. The combined laser beams are coupled into an inverse laser scanning microscope (LSM 510, Zeiss, Jena, Germany) and focused onto the tissue section with a 20 × objective (Plan-Apochromat, NA 0.8, Zeiss, Germany). CARS, SHG, and TPEF signals are simultaneously detected by photomultiplier tubes (PMT, Hamamatsu Photonics, Hamamatsu, Japan) in the forward direction (CARS, SHG), and the backward direction (TPEF). CARS visualizes the distribution of methylene groups (CH_2_), which are abundant in lipids at the Raman resonance of 2850 ${\mathrm{cm}}^{-1}$, and the signal was detected using a 550 nm bandpass filter. SHG is a scattering effect that occurs in structures that lack inversion symmetry, such as collagen fibers collected at 415 nm (bandpass 415/3, Omega Optical, USA). At a wavelength of 435–485 nm, TPEF visualizes the skin’s autofluorophores^57^, which are detected at 458 nm (shortpass 650 nm, bandpass 458∕64 nm, Semrock). Alternatively, the fluorescence signal can be reflected onto a hybrid GaAsP detector (HPM-100-40, Becker & Hickl, Germany) by a dichroic mirror (600 nm shortpass) and filtered by a shortpass 650 nm and bandpass 458∕64 nm (Semrock) filter for 2P-FLIM imaging. A time-correlated single-photon counting (TCSPC) system (SPC-150, Becker & Hickl, Germany) was used in determining the fluorescence lifetime. The imaging parameters for 2P-FLIM measurements include 512 × 512 pixels, 1024 time channels, and a pixel dwell time of 1.6 μs. To keep the FLIM detector’s count rate below 1, the power was adjusted to approximately 10 mW for the pump beam and 30 mW for the Stokes beam. To acquire the multimodal images, large area scans with a resolution of 1024 × 1024 pixels, a frame average of 8, and a pixel dwell time of 1.6 μs were recorded. To avoid photodamage, the laser power was adjusted to 50 mW for both the pump and Stokes beams at the sample. The photodamage thresholds and parameters for average power and peak irradiance have already been discussed^56,58^. In addition, 3-dimensional (3D) images were obtained by recording z-stacks of 20 μm stack size with a step size of 0.5 μm. Scans of 450 × 450 μm were recorded for the 20 × objective (Plan-Apochromat, NA 0.8, Zeiss, Germany) and 142 × 142 μm for the 63× objective (Plan-Apochromat, NA 1.4, Zeiss, Germany).

### Immunohistochemistry

For the evaluation of the epithelial barrier disruption and identification of the immune cells in lamina propria, Alexa Fluor 647 mouse anti-E-Cadherin antibody (1:100, BD Biosciences, USA), an antibody Alexa Fluor 594 anti-human CD11b (1:300, BioLegend, USA) and DAPI (4′,6-diamidino-2-phenylindole) Fluoromount-G (SouthernBiotech, USA) were used (Fig. 3A and C). The microscopic images were performed under an Axio Observer Z.1 Microscope (Zeiss, Germany) using a Plan-Apochromat 40×/0.95 Korr M27 objective. Data analysis was performed using the Zeiss ZEN 2.3 (blue edition) software.

### Hematoxylin–eosin (H&E) staining of tissue sections

The tissue sections after multimodal imaging were stained with hematoxylin and eosin (H&E). The tissue sections were dehydrated using the ethanol series of 70%, 80%, and 90% followed by 100% ethanol washes. Dehydrated tissue sections were stained with hematoxylin, followed by washing with distilled water. The hematoxylin-stained tissue sections were further stained with eosin followed by washing in distilled water. The slides were dried in an incubator and stored at room temperature. The brightfield images of H&E stained tissue were collected using an Olympus microscope (Olympus, Germany) with a 40×/0.8 objective.

### Transmission electron microscopy (TEM)

TEM samples were prepared by tissue fixation with 4% glutaraldehyde for 2 h. After that, the tissue was transferred to phosphate-buffered saline (PBS). Post-fixation was performed in a 1% osmium tetroxide solution for another 2 h. The tissue sample was washed twice in fresh PBS before dehydration in a graded ethanol/water series. After dehydration, resin infiltration was performed with Embed812 with 18 µl of DMP-30 added as an initiator. For proper infiltration, the resin was first applied in a 1:3 Embed 812:EtOH diluted resin solution for 1 h, then a 1:1 mixture for 2 h, and finally a 3:1 mixture overnight at room temperature. Finally, samples were infiltrated with 100% Embed/DMP-30 solution for another 24 h. The tissue was then transferred to a BEEM capsule (Plano, Germany), and fresh, undiluted Embed/DMP-30 was added. The samples were cured at 60 °C for 12 h.

Thin slices of 80 nm size of the embedded tissue sample were prepared with an RMC Ultramikrotom PowerTome PT XL (Reichert, Germany) by using a diamond knife (Diatome, Germany). Slices were deposited onto carbon-coated TEM grids (200 mesh, Quantifoil, Germany) and additional staining with uranylless stain (1.5 min, Electron Microscopy Service (EMS)) and lead citrate (1 min, Electron Microscopy Services (EMS)) were performed.

TEM measurements of the stained tissue samples were performed on an FEI Tecnai G^2^ 20 (FEI, The Netherlands) at an acceleration voltage of 200 kV utilizing a LaB_6_ filament. Images were acquired with a MegaView CCD camera (1376 × 1024 pixels, Olympus Soft Imaging Solutions (OSIS)) or a 4096 × 4096 pixel FEI Eagle CCD camera. The image analysis and contrast adjustment were performed utilizing ImageJ 1.47 V.

### Ethics approval and consent to participate

The study was approved by the committee of human ethics (3285–10/11) at Jena University Hospital, Jena, Germany. Informed consent was obtained from all the patients.

## Supplementary Information

Supplementary Figures.

## Data Availability

The datasets generated during and/or analyzed during the current study are available from the corresponding author on reasonable request.

## Abbreviations

AJC: Apical junction complex
CARS: Coherent anti-Stokes Raman-scattering
CLE: Confocal endomicroscopy
TPEF: Two-photon excited autofluorescence
SHG: Second harmonic generation
2P-FLIM: Two-photon fluorescence lifetime microscopy
TEM: Transmission electron microscope
H&E: Hematoxylin and eosin
IHC: Immunohistochemistry
UC: Ulcerative colitis, IBD: Inflammatory bowel diseases
NAD(P)H: Nicotinamide adenine dinucleotide phosphate
