# Supplementary material for: Intestinal epithelial barrier integrity investigated by label-free techniques in ulcerative colitis patients
Source: Sci Rep. 2023 Feb 15;13:2681. doi: 10.1038/s41598-023-29649-y (PMC9931702; doi:10.1038/s41598-023-29649-y)
Supplement: Supplementary file 1 — Supplementary Figures. [file 41598_2023_29649_MOESM1_ESM.docx]

**Intestinal epithelial barrier integrity investigated by label-free techniques in ulcerative colitis patients**

Elsie Quansah+^1,2^, Elena Gardey+^3,4^, Anuradha Ramoji+^,1,2,5*^, Tobias Meyer-Zedler^1,2^, Bianca Goehrig^6^, Astrid Heutelbeck^6^_,_ Stephanie Hoeppener^4,7^, Michael Schmitt^1,2^, Maximilian Waldner^8^, Andreas Stallmach^3^ and Jürgen Popp^1,2^.

^1^Friedrich Schiller University Jena, Institute of Physical Chemistry (IPC) and Abbe Center of Photonics (IPC), Member of the Leibniz Centre for Photonics in Infection Research (LPI), Helmholtzweg 4, -07743 Jena, Germany

^2^Leibniz Institute of Photonic Technology (IPHT), Member of Leibniz Health Technologies, Member of the Leibniz Centre for Photonics in Infection Research (LPI), Albert-Einstein-Straße 9, 07745 Jena, Germany

^3^Jena University Hospital, Department of Internal Medicine IV (Gastroenterology, Hepatology, Infectious Diseases and Interdisciplinary Endoscopy), Friedrich Schiller University Jena, Am Klinikum 1, 07747 Jena, Germany

^4^Friedrich Schiller University Jena, Jena Center for Soft Matter (JCSM), Philosophenweg 7, 07743 Jena, Germany

^5^Jena University Hospital, Center for Sepsis Control and Care (CSCC), Friedrich Schiller University Jena, Erlanger Allee 101, 07747 Jena, Germany

^6^Jena University Hospital, Institute for occupational, social, and environmental medicine, Am Klinikum 1, 07747, Jena, Germany

^7^Friedrich Schiller University Jena, Laboratory of Organic and Macromolecular Chemistry (IOMC), Humboldtstraße 10, 07743 Jena, Germany

^8^Department of Medicine, University of Erlangen-Nuremberg, 91054 Erlangen, Germany.

*+Equal contributions*

**Corresponding author* [*anuradha.ramoji@uni-jena.de*](mailto:anuradha.ramoji@uni-jena.de)*, Tel. Nr. 03641 9390926*

Supporting Information


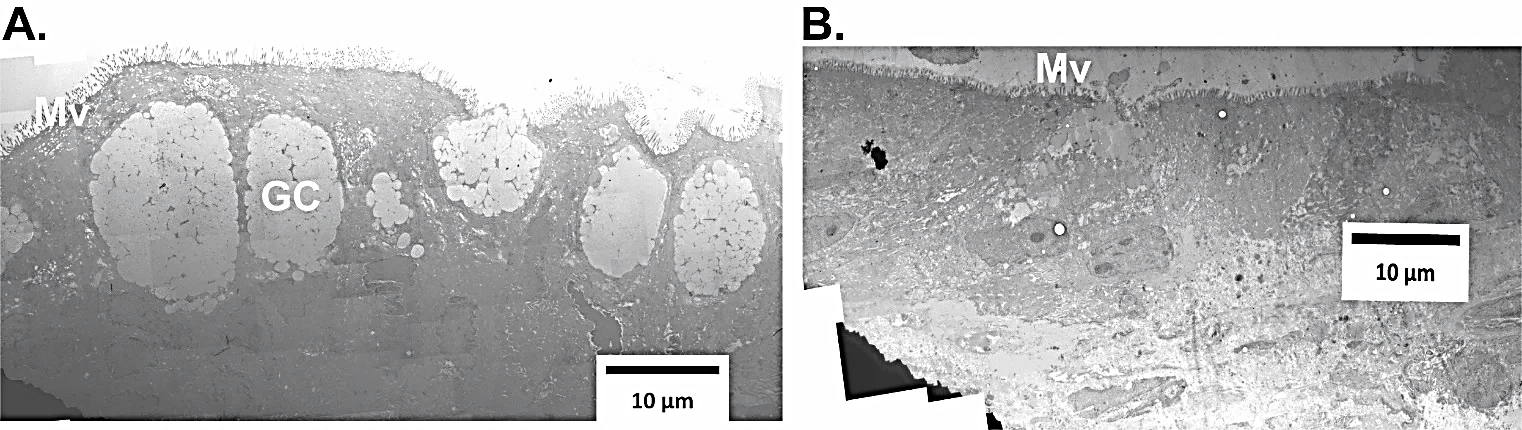


**Figure S1**. Stitched transmission electron microscopy (TEM) images of healthy (**A**) and inflamed (**B**) colonic mucosa. Several intact microvilli (Mv) and goblet cells (GC) are observed in the healthy tissue in contrast to the reduced goblet cells in the inflamed tissue.


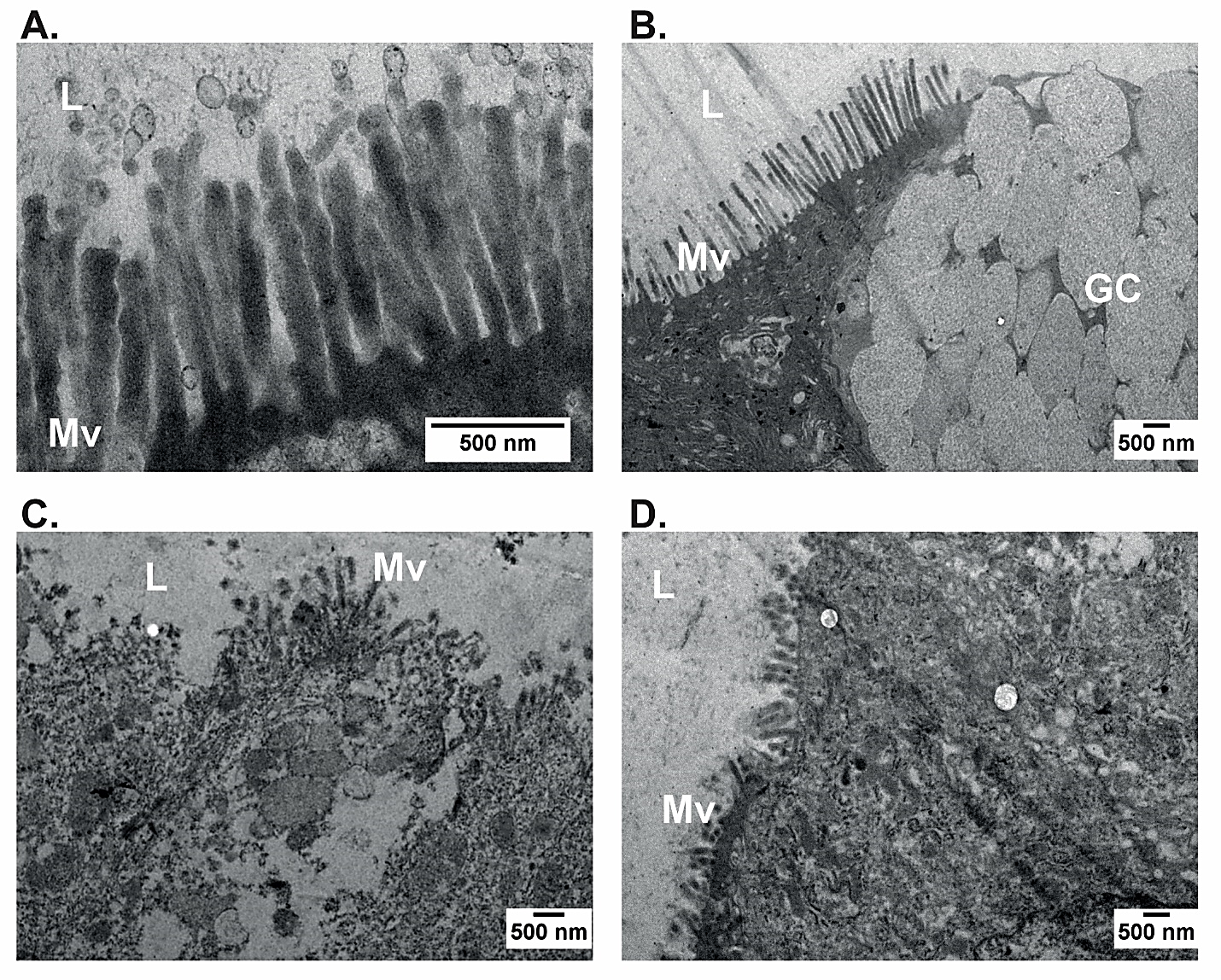
**Figure S2**. The microvilli (Mv) in the healthy tissue (**A, B**) are long and intact, while those in the inflamed tissue (**C**, **D**) are disturbed.

L – Lumen, GC – Goblet cells.
